# Editorial: Binge Drinking in the Adolescent and Young Brain

**DOI:** 10.3389/fpsyg.2018.02724

**Published:** 2019-01-09

**Authors:** Eduardo López-Caneda, Fernando Cadaveira, Salvatore Campanella

**Affiliations:** ^1^Psychological Neuroscience Lab, Research Center in Psychology (CIPsi), School of Psychology, University of Minho, Braga, Portugal; ^2^Department of Clinical Psychology and Psychobiology, University of Santiago de Compostela, Galicia, Spain; ^3^Laboratoire de Psychologie Médicale et d'Addictologie, ULB Neuroscience Institute, CHU Brugmann-Université Libre de Bruxelles, Brussels, Belgium

**Keywords:** alcohol, binge drinking, adolescence, cognitive function, brain

Alcohol is considered the world's third largest risk factor for disease and about 6% of all deaths worldwide are attributable to this substance (Rehm et al., 2009; World Health Organization, 2014). Excessive alcohol use is especially harmful for younger age groups, where alcohol has been (directly or indirectly) related to more than 30% of deaths among males aged 15–29 years in the American and European regions (World Health Organization, 2011).

Binge drinking (BD), an excessive but episodic alcohol consumption pattern, has become a major public health problem as it is held accountable for multiple adverse consequences, including poor quality of life, injuries, risky sexual behavior and neurocognitive deficits (White and Hingson, 2013; Carbia et al., 2018; Dormal et al., 2018). This pattern, defined as the consumption of 5 or more drinks (male) or 4 or more drinks (female) in about 2 h (National Institute of Alcohol Abuse Alcoholism, 2004), is a regular practice in about one third of European and American youths (Kraus et al., 2016 Substance Abuse and Mental Health Services Substance Abuse and Mental Health Services Administration, 2016). The high prevalence of BD at this age is of particular concern since adolescence and youth are in a period of special vulnerability to neurotoxic effects of alcohol, mainly due to the structural and functional changes going on in the brain throughout this key developmental stage (Jones et al., 2018).

Research on this topic has significantly increased in recent years. As such, the number of studies involving BD during adolescence and youth have almost quintupled during the period 2004–2014 (from 111 in 2004 to 510 in 2014), with a slight increase in the last few years (see Figure 1). The objective of this Research Topic was to produce and compile a highly informative collection of original research and reviews aiming at cover a comprehensive framework of aspects related to BD from different domains (animal and human), perspectives (cellular, behavioral, neuropsychological, neuroimaging, etc.), and methods (e.g., biochemical, behavioral, psychophysiological, neurostructural, and neurofunctional).

**Figure 1:**
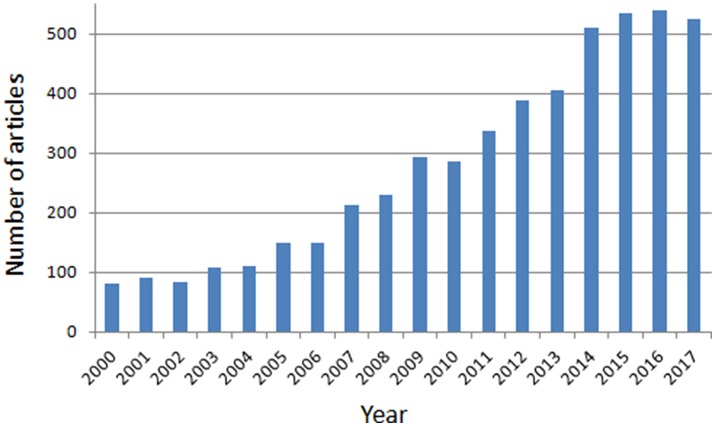
Number of articles involving binge drinking during adolescence and youth for the period 2000–2017. The search strategy was conducted in PubMed with the following key terms: [(“binge drinking” OR “binge drinkers” OR “heavy drinking” OR “heavy drinkers” OR “heavy episodic drinking” OR “college drinking” OR “college drinkers” OR “social drinkers”) AND (adolescen^*^ OR youth^*^ OR teen^*^ OR “young” OR “young adults” OR “college students” OR “university students”].

With regard to animal studies, two articles included in this Research Topic involved animal models of BD. Lee et al. provided novel evidence that BD during adolescence induces profound negative affect (anxiety- and depressive-like behaviors), excessive alcohol consumption and dysregulation within extended amygdala structures, which manifest during protracted withdrawal in adulthood. Nickell et al., in turn, studied hippocampal neurogenesis in adolescent male rats exposed to BD. They observed a marked increment in cell proliferation in hippocampus following 4-day alcohol exposure, although it is not clear whether this reactive neurogenesis is a beneficial repair mechanism (e.g., recovery of hippocampal structure and function) or a pathological phenomenon (e.g., reflecting ectopic new neurons as that observed in seizure models).

Within human studies, Cortés-Tomás et al. developed a new abbreviated version of the Alcohol Use Disorder Identification Test (AUDIT) which includes the combination of items 2 and 3 in a revised form. Their findings revealed that using of these two revised items lead to a more precise identification of BD in adolescents. Using the classical AUDIT questionnaire, Gómez et al. indentified five different profiles of Spanish university students based on their alcohol use over 9 years and reported a generalized reduction of the AUDIT scores over this period for all profiles, suggesting a common effect of “maturing out” of problematic alcohol use in their late twenties. Pilatti et al. observed, in a large sample of Argentinean college freshmen, a high prevalence of BD in this country (around 55% of college students between 18 and 30 years old reported BD in the last 6 months). In addition, alcohol was the entry-point for the consumption of tobacco and marijuana and an early drinking onset was associated with greater use of alcohol.

Adan et al. reviewed the findings on personality traits related to binge drinkers (BDs) and conclude that the main characteristics of personality related to the practice of BD were impulsivity and high sensation seeking, as well as anxiety sensitivity, neuroticism, extraversion and conscientiousness. Dir et al., in turn, provided an overview of potential gender differences in risk factors for adolescent BD. They showed that—presumably due to the sex-specific neurobiological changes that occur during adolescent development—there is a differential risk for BD between males and females. Thus, while the main factors contributing for BD in females were stress, depression, and other internalizing behaviors, the most significant contributions for risk of BD in males were driven by externalizing symptoms such as behavioral disinhibition, impulsivity and sensation seeking. In the same vein, in an online cross-sectional study with more than 1,800 French students, Rolland et al. revealed that severity of BD was associated with, among other factors, male gender, younger age and sensation seeking. In addition, they pointed out that BD score was correlated with severity of binge eating (BE), but not with other disordered eating symptoms, indicating that BD and BE may share common characteristics, including an impaired emotion regulation.

The studies by Lannoy et al. and Poncin et al. explored emotional processing and emotion regulation strategies in young BDs, respectively. Results of Lannoy et al. showed no significant differences between the control and BD groups in emotional processing abilities as measured by an emotional crossmodal task. Similarly, Poncin et al. did not find differences between BDs and controls in the overall scores of emotional distress induced by an insoluble anagrams task, though emotional distress was related to more self-blame, rumination, and maladaptive regulation strategies in BDs only.

Amid the neuropsychological studies that evaluated cognitive functions, Peeters et al. examined whether the imbalance between behavioral control and reward sensitivity might account for risky behaviors such as alcohol and cannabis use. They found that a weak effortful control in early adolescence (age 11) was a significant unique predictor of risk taking behavior in mid adolescence (age 16), particularly among adolescents who were more reward sensitive. In the same vein, Bø et al. reported that future severity of BD was associated with making risky decisions in the prospect of gain in the Information Sampling task, which was suggestive of reward hypersensitivity in young BDs. However, in a 4 years follow-up study conducted by Carbia et al., adolescents and young adults with a BD pattern did not show deficits in decision making under ambiguous conditions as measured by the Iowa gambling task, though there were gender-related differences in task performance as females displayed a higher sensitivity to loss frequency than males.

Gil-Hernandez et al. in a study that covered a wide range of cognitive functions (e.g., working memory, inhibition, cognitive flexibility, self-control) and ages (13–15, 16–18, and 19–22 years), observed that control subjects obtained better results than BDs but only in the 19–22-year-old range, suggesting that several years of BD history are necessary to make cognitive impairments apparent through neuropsychological tests.

Also by means of neuropsychological tasks, Vinader-Caerols et al. assessed the effects of different blood alcohol concentrations (BAC) on memory in adolescents with a history of BD. Both immediate visual memory and working memory were susceptible of being impaired by high doses of alcohol, but only immediate visual memory was affected by moderate doses of alcohol, suggesting that immediate visual memory is more sensitive than working memory to the neurotoxic effects of alcohol in adolescent BDs. These results are in line with the mini-review conducted by Hermens and Lagopoulos, who compiled numerous studies on the detrimental effects of BD in learning and memory (and on its main structural support, the hippocampus) and, particularly, on the ability of this pattern of consumption to induced memory loss (blackouts) in the adolescent and young adult population.

Besides the neuropsychological studies, two articles of this Research Topic used electroencephalography (EEG) for exploring the relationship between BD and memory deficits. The Smith et al.'s study analyzed brain electrical activity by event-related potentials (ERPs) while participants (BDs, cannabis users and controls) completed a modified verbal learning task. The authors showed that BDs displayed larger P540 amplitude (an electrophysiological index of recollection) relative to controls, suggesting greater use of recollection-based recognition in the BD group. The study by Folgueira-Ares et al. used the ERP technique to explore potential differences between young BDs and controls during the memory encoding process in a face-name associative memory task. While the control group displayed larger ERP amplitudes during memory encoding for subsequently remembered face-name pairs than for subsequently forgotten pairs, BDs exhibited similar neural activity for successful and unsuccessful encoding, presumably reflecting a neural signature of BD-induced impairment on this memory stage.

Cservenka and Brumback reviewed neuroimaging research involving the effects of BD on adolescent and young adult brain structure and function. In their mini-review, authors highlighted that most of the neurostructural studies seem to point to reduced prefrontal cortex (PFC) and cerebellar volume as well as to attenuated white matter development in young people with a BD pattern. Additionally, they also report that BDs usually show greater reliance on fronto-parietal regions while performing cognitive tasks linked to working memory, verbal learning, and inhibitory control processes. However, and in line with the authors' assertion that additional replication studies are needed in order to verify the direction of BD-induced brain abnormalities, two studies of the present Research Topic suggest a different profile of such anomalies. Indeed, Sousa et al.'s study reported increased gray matter densities in the left middle frontal gyrus in BDs, when compared with alcohol abstinent controls. Similarly, the study by Cohen-Gilbert et al. showed that a higher recent incidence of BD was associated with decreased activation of PFC regions during negative relative to neutral inhibitory trials in an emotional Go/NoGo task. These apparently inconsistent results should encourage researchers to perform greater efforts in terms of homogenization of sample selected (e.g., age, inclusionary criteria for BD and control groups), tasks chosen (e.g., identical tasks for replication of neurofunctional findings) and type of analysis conducted (e.g., volume, thickness or density when analyzing brain morphology).

Collectively, this Research Topic contains a compendium of articles that address multiple aspects of BD during adolescence and young adulthood (such as identification, prevalence, gender differences, neurocognitive consequences, etc.). We hope that this collection exhorts researchers to extend and refine the studies conducted to date as well as to address still unanswered questions. In this regard, attention should be paid to the impact of particularly high levels of alcohol consumption, namely high-intensity drinking (Patrick and Azar, 2018). Likewise, follow-up studies should be carried out to shed light on the causes and consequences of BD and on the course of problems/improvements observed with the maintenance/cessation of this pattern (Maurage et al., 2009; López-Caneda et al., 2014; Winward et al., 2014; Carbia et al., 2017). It is also important to assess the role of possible interactions with other substances—illegal or prescription drugs—(Blazer and Wu, 2009; Keith et al., 2015) as well as to clarify whether there are gender differences and how they may be related to the different neuromaturational pace that occurs during this developmental period (Medina et al., 2008; Squeglia et al., 2015). Finally, further research should also extend studies—which at present are almost entirely limited to university students—to the general population.

In short, these, among many other issues, should raise awareness of the importance of addressing pending challenges in this—still relatively new—research field and, therefore, encourage the growth of a strong and more comprehensive body of knowledge that can be translated into measurable societal impact.

## Author Contributions

EL-C conceptualized the proposal and wrote the first draft of the manuscript. All authors read, revised, and approved the final manuscript.

### Conflict of Interest Statement

The authors declare that the research was conducted in the absence of any commercial or financial relationships that could be construed as a potential conflict of interest.
